# Supplementary material for: Integrated Serosurveillance of Infectious Diseases Using Multiplex Bead Assays: A Systematic Review
Source: Trop Med Infect Dis. 2025 Jan 10;10(1):19. doi: 10.3390/tropicalmed10010019 (PMC11769346; doi:10.3390/tropicalmed10010019)
Supplement: Supplementary file 1 [file tropicalmed-10-00019-s001.zip › tropicalmed-3411107-supplementary.pdf]

S1: Search results

Database and Number of Hits	Boolean
Pubmed = 429	("multiplex bead assay*" OR "MBA" OR "Luminex assay" OR "multiplex antigen assay" OR "bead assay" OR "multiplex flow immunoassay" OR "multiplex assay") AND ("neglected tropical disease*" OR NTD OR "vaccine preventable disease*" OR VPD OR arbovirus OR "infectious disease*" OR "Buruli ulcer" OR Chagas OR dengue OR chikungunya OR dracunculiasis OR "Guinea worm" OR echinococcosis OR trematodiases OR "human African trypanosomiasis" OR "sleeping sickness" OR leishmaniasis OR leprosy OR "Hansen's disease" OR lymphatic filariasis OR "LF" OR mycetoma OR chromoblastomycosis OR onchocerciasis OR "river blindness" OR podoconiosis OR rabies OR scabies OR schistosomiasis OR "soil transmitted helminth*" OR STH OR taeniasis OR cysticercosis OR trachoma OR yaws OR poliomyelitis OR measles OR rubella OR cholera OR tetanus OR Diphtheria OR "Haemophilus influenza type b" OR HIB OR Pertussis OR "Pneumococcal disease" OR Rotavirus OR varicella OR malaria OR plasmodium OR zika) AND (epidemiology OR monitor* OR serolog* OR surv* OR prevalence*)
Medline = 511	("multiplex bead assay*" OR "MBA" OR "Luminex assay" OR "multiplex antigen assay" OR "bead assay" OR "multiplex flow immunoassay" OR "multiplex assay") AND ("neglected tropical disease*" OR NTD OR "vaccine preventable disease*" OR VPD OR arbovirus OR "infectious disease*" OR "Buruli ulcer" OR Chagas OR dengue OR chikungunya OR dracunculiasis OR "Guinea worm" OR echinococcosis OR trematodiases OR "human African trypanosomiasis" OR "sleeping sickness" OR leishmaniasis OR leprosy OR "Hansen's disease" OR lymphatic filariasis OR "LF" OR mycetoma OR chromoblastomycosis OR onchocerciasis OR "river blindness" OR podoconiosis OR rabies OR scabies OR schistosomiasis OR "soil transmitted helminth*" OR STH OR taeniasis OR cysticercosis OR trachoma OR yaws OR poliomyelitis OR measles OR rubella OR cholera OR tetanus OR Diphtheria OR "Haemophilus influenza type b" OR HIB OR Pertussis OR "Pneumococcal disease" OR Rotavirus OR varicella OR malaria OR plasmodium OR zika) AND (epidemiology OR monitor* OR serolog* OR surv* OR prevalence*)
Scopus = 3355	("multiplex bead assay*" OR "MBA" OR "Luminex assay" OR "multiplex antigen assay" OR "bead assay" OR "multiplex flow immunoassay" OR "multiplex assay") AND ("neglected tropical disease*" OR ntd OR "vaccine preventable disease*" OR vpd OR arbovirus OR "infectious disease*" OR "Buruli ulcer" OR chagas OR dengue OR chikungunya OR dracunculiasis OR "Guinea worm" OR echinococcosis OR trematodiases OR "human African trypanosomiasis" OR "sleeping sickness" OR leishmaniasis OR leprosy OR "Hansens disease" OR lymphatic AND filariasis OR "LF" OR mycetoma OR chromoblastomycosis OR onchocerciasis OR "river blindness" OR podoconiosis OR rabies OR scabies OR schistosomiasis OR "soil transmitted helminth*" OR sth OR taeniasis OR cysticercosis OR trachoma OR yaws OR poliomyelitis OR measles OR rubella OR cholera OR tetanus OR diphtheria OR "Haemophilus influenza type b" OR hib OR pertussis OR "Pneumococcal disease" OR rotavirus OR varicella OR malaria OR plasmodium OR zika) AND (epidemiology OR monitor* OR serolog* OR surv* OR prevalence*)
Embase = 448	("multiplex bead assay*" OR "MBA" OR "Luminex assay" OR "multiplex antigen assay" OR "bead assay" OR "multiplex flow immunoassay" OR "multiplex assay") AND ("neglected tropical disease*" OR ntd OR "vaccine preventable disease*" OR vpd OR arbovirus OR "infectious disease*" OR "Buruli ulcer" OR chagas OR dengue OR chikungunya OR dracunculiasis OR "Guinea

	worm" OR echinococcosis OR trematodiases OR "human African trypanosomiasis" OR "sleeping sickness" OR leishmaniasis OR leprosy OR "Hansens disease" OR lymphatic AND filariasis OR "LF" OR mycetoma OR chromoblastomycosis OR onchocerciasis OR "river blindness" OR podoconiosis OR rabies OR scabies OR schistosomiasis OR "soil transmitted helminth*" OR sth OR taeniasis OR cysticercosis OR trachoma OR yaws OR poliomyelitis OR measles OR rubella OR cholera OR tetanus OR diphtheria OR "Haemophilus influenza type b" OR hib OR pertussis OR "Pneumococcal disease" OR rotavirus OR varicella OR malaria OR plasmodium OR zika) AND (epidemiology OR monitor* OR serolog* OR surv* OR prevalence*)
Cochrane = 22 trials	("multiplex bead assay*" OR "MBA" OR "Luminex assay" OR "multiplex antigen assay" OR "bead assay" OR "multiplex flow immunoassay" OR "multiplex assay") AND ("neglected tropical disease*" OR ntd OR "vaccine preventable disease*" OR vpd OR arbovirus OR "infectious disease*" OR "Buruli ulcer" OR chagas OR dengue OR chikungunya OR dracunculiasis OR "Guinea worm" OR echinococcosis OR trematodiases OR "human African trypanosomiasis" OR "sleeping sickness" OR leishmaniasis OR leprosy OR "Hansens disease" OR lymphatic AND filariasis OR "LF" OR mycetoma OR chromoblastomycosis OR onchocerciasis OR "river blindness" OR podoconiosis OR rabies OR scabies OR schistosomiasis OR "soil transmitted helminth*" OR sth OR taeniasis OR cysticercosis OR trachoma OR yaws OR poliomyelitis OR measles OR rubella OR cholera OR tetanus OR diphtheria OR "Haemophilus influenza type b" OR hib OR pertussis OR "Pneumococcal disease" OR rotavirus OR varicella OR malaria OR plasmodium OR zika) AND (epidemiology OR monitor* OR serolog* OR surv* OR prevalence*) in All Text - (Word variations have been searched)
Total = 4765	Loaded into Endnote 29.11.23<br>Duplicates removed by Endnote (n=818) 29.11.23<br>Loaded into Covidence 29.11.23<br>Duplicates removed by Covidence 138<br>Total to screen = 3,809

S2: Pathogens studied

Diseases Group	*Species*	Studies	Count
Malaria	*Plasmodium falciparium*	Arzika et al., 2022; Assefa et al., 2019; Byrne et al., 2022; Byrne et al., 2023; Chan et al., 2022; Feleke et al., 2019; Herman et al., 2023; Jeang et al., 2023; Khaireh et al., 2012; Koffi et al., 2017; Labadie-Bracho, van Genderen, & Adhin, 2020; Leonard et al., 2022; Lu et al., 2020; Macalinao et al., 2023; McCaffery et al., 2022; Monteiro et al., 2021; Moss et al., 2011; Njenga et al., 2020; Oviedo et al., 2022; Perraut et al., 2017; Plucinski et al., 2018; Poirier et al., 2016; Priest et al., 2016; Rogier et al., 2017; Rogier et al., 2019; van den Hoogen et al., 2021	26
	Plasmodium vivax	Arzika et al., 2022; Assefa et al., 2019; Byrne et al., 2022; Byrne et al., 2023; Feleke et al., 2019; Herman et al., 2023; Jeang et al., 2023; Khaireh et al., 2012; Labadie-Bracho, van Genderen, & Adhin, 2020; Leonard et al., 2022; Lu et al., 2020; Macalinao et al., 2023; McCaffery et al., 2022; Monteiro et al., 2021; Oviedo et al., 2022; Plucinski et al., 2018; Priest et al., 2016; Rogier et al., 2017; Rogier et al., 2019	19
	Plasmodium ovale	Arzika et al., 2022; Assefa et al., 2019; Feleke et al., 2019; Herman et al., 2023; Plucinski et al., 2018	5
	Plasmodium malariae	Arzika et al., 2022; Assefa et al., 2019; Byrne et al., 2023; Feleke et al., 2019; Herman et al., 2023; Koffi et al., 2017; Labadie-Bracho, van Genderen, & Adhin, 2020; Lu et al., 2020; Monteiro et al., 2021; Njenga et al., 2020; Oviedo et al., 2022; Perraut et al., 2017; Plucinski et al., 2018; Rogier et al., 2019; van den Hoogen et al., 2021	15
	Plasmodium knowlesi	Byrne et al., 2023	1
Lymphatic Filariasis	*Wuchereria bancrofti*	Cadavid Restrepo et al., 2022; Chan et al., 2022a; Chan et al., 2022b; Fornace et al., 2022; Fujii et al., 2014; Njenga et al., 2020; Plucinski et al., 2018; Priest et al., 2016; Won et al., 2018	9
	Brugia malayi	Cadavid Restrepo et al., 2022; Chan et al., 2022a; Chan et al., 2022b; Kwan et al., 2018; Njenga et al., 2020; Plucinski et al., 2018; Poirier et al., 2016; Priest et al., 2016; Won et al., 2018	9
Strongyloidiasis	*Strongyloides stercoralis*	Chan et al., 2022a; Chan et al., 2022b; Fornace et al., 2022; Kwan et al., 2018; Njenga et al., 2020; Plucinski et al., 2018; Priest et al., 2016	7
Dengue virus		Chan et al., 2022a; Poirier et al., 2016	2

Chikungunya virus		Chan et al., 2022a; Poirier et al., 2016	2
Schistosomiasis	*Schistosoma masoni*	Fornace et al., 2022; Njenga et al., 2020	2
Trachoma	*Chlamydia trachomatis*	Aiemjoy et al., 2020; Chan et al., 2022a; Chan et al., 2022b; Cooley et al., 2021; Fornace et al., 2022; Mentzer et al., 2022	6
Yaws	*Treponematoses pallidum*	Chan et al., 2022a; Chan et al., 2022b; Cooley et al., 2021; Fornace et al., 2022	4
Onchocerciasis	*Onchocerca volvulus*	Fornace et al., 2022	1
Leishmaniasis	*Leishmania donovani*	Fujii et al., 2014	1
Cysticercosis	*Taenia solium*	Priest et al., 2016	1
Diphtheria	*Corynebacterium diphtheriae*	Boey et al., 2021; Breakwell et al., 2020; Caboré, Piérard, & Huygen, 2016; Feldstein et al., 2020; Khetsuriani et al., 2022; Minta et al., 2020; Njenga et al., 2020; Tohme et al., 2023	8
Tetanus	*Clostridium tetani*	Boey et al., 2021; Breakwell et al., 2020; Caboré, Piérard, & Huygen, 2016; Feldstein et al., 2020; Khetsuriani et al., 2022; Minta et al., 2020; Njenga et al., 2020; Tohme et al., 2023	8
Pertussis	*Bordetella pertussis*	Boey et al., 2021; Caboré, Piérard, & Huygen, 2016	2
Rubella	*Rubella virus*	Breakwell et al., 2020; Feldstein et al., 2020; Minta et al., 2020	3
Measles	*Measles virus*	Breakwell et al., 2020; Feldstein et al., 2020; Minta et al., 2020; Njenga et al., 2020	4
Varicella zoster	*Varicella zoster virus*	Mentzer et al., 2022	1
Giardia	*Giardia lamblia*	Arnold et al., 2019; Arzika et al., 2022; Fornace et al., 2022; Zambrano et al., 2017	4
	Giardia dudenalis	Chan et al., 2022b; Mosites et al., 2018	2
	Giardia intestinalis	Aiemjoy et al., 2020; Miernyk et al., 2019	2
	Campylobacter jejuni	Aiemjoy et al., 2020	1
Bacterial intestinal infections	*enterotoxigenic Escherichia Coli*	Aiemjoy et al., 2020; Arnold et al., 2019; Arzika et al., 2022; Chan et al., 2022a; Zambrano et al., 2017	5
	Salmonella species	Aiemjoy et al., 2020; Arnold et al., 2019; Arzika et al., 2022	3
	Vibrio cholera	Aiemjoy et al., 2020; Arnold et al., 2019; Fujii et al., 2014; Zambrano et al., 2017	4
	Helicobacter pylori	Mentzer et al., 2022	1
Other bacterial	*Campylobacter species*	Arnold et al., 2019; Arzika et al., 2022; Zambrano et al., 2017	3
	Group A streptococcus	Arzika et al., 2022	1

Parasitic intestinal infections	*Entamoeba histolytica*	Aiemjoy et al., 2020; Arnold et al., 2019; Chan et al., 2022a; Fujii et al., 2014; Zambrano et al., 2017	5
	Toxoplasma gondii	Chan et al., 2022a; Chan et al., 2022b; Fujii et al., 2014; Mentzer et al., 2022; Priest et al., 2016	5
	Echinococcus granulosus	Miernyk et al., 2019	1
Cryptosporidium	*Cryptosporidium parvum*	Aiemjoy et al., 2020; Arnold et al., 2019; Arzika et al., 2022; Chan et al., 2022a; Miernyk et al., 2019; Mosites et al., 2018; Zambrano et al., 2017	7
Respiratory viruses	SARS-CoV-2	Woudenberg et al., 2021	1
	Human coronaviruses	Woudenberg et al., 2021	1
Intestinal virus	Norovirus	Arnold et al., 2019; Zambrano et al., 2017	2
Herpes virus family	Epstein Barr Virus	Mentzer et al., 2022	1
	Herpes Simplex Virus	Mentzer et al., 2022	1
	Cytomegalovirus virus	Mentzer et al., 2022	1
	Kaposi's sarcoma virus	Mentzer et al., 2022	1
	Human Herpes Virus	Mentzer et al., 2022	1
Other viruses	Hepatitis B & C Virus	Mentzer et al., 2022	1
	Human Immunodeficiency Virus	Fujii et al., 2014; Mentzer et al., 2022	2
	T-lymphotropic virus	Mentzer et al., 2022	1
	Papilloma virus	Mentzer et al., 2022	1
	JC Virus	Mentzer et al., 2022	1
	BK Virus	Mentzer et al., 2022	1

S3: Antigens

Disease	Species	Antigens	Studies
Malaria	*P. falciparium*	PfMSP1-19	(Arzika et al., 2022; Assefa et al., 2019; Byrne et al., 2022; Byrne et al., 2023; Y. Chan et al., 2022; Feleke et al., 2019; Herman et al., 2023; Khaireh et al., 2012; Koffi et al., 2017; Labadie-Bracho, van Genderen, & Adhin, 2020; Leonard et al., 2022; Lu et al., 2020; Macalinao et al., 2023; McCaffery et al., 2022; Monteiro et al., 2021; Moss et al., 2011; Njenga et al., 2020; Oviedo et al., 2022; Perraut et al., 2017; Plucinski et al., 2018; Poirier et al., 2016; Priest et al., 2016; Rogier et al., 2018; van den Hoogen et al., 2021)
		PfMSP1-42	(Jeang et al., 2023; Poirier et al., 2016; Priest et al., 2016; Rogier et al., 2017)
		PfMSP1-19(FVO)	(Poirier et al., 2016; Priest et al., 2016)
		PfMSP1-42(belem)	(Priest et al., 2016)
		PfMSP4-20	(Koffi et al., 2017)
		PfCSP	(Arzika et al., 2022; Feleke et al., 2019; Jeang et al., 2023; Koffi et al., 2017; Labadie-Bracho, van Genderen, & Adhin, 2020; Leonard et al., 2022; Lu et al., 2020; Perraut et al., 2017; Plucinski et al., 2018; Rogier et al., 2018)
		PfLSA-1	(Arzika et al., 2022; Feleke et al., 2019; Labadie-Bracho, van Genderen, & Adhin, 2020; Leonard et al., 2022; Lu et al., 2020; Plucinski et al., 2018; Rogier et al., 2018)
		PfLSA1-41	(Koffi et al., 2017; Perraut et al., 2017)
		PfLSA3	(Koffi et al., 2017)
		PfMSP1-29	(Njenga et al., 2020)
		PfPTP3	(Moss et al., 2011)
		PfAMA1	(Arzika et al., 2022; Assefa et al., 2019; Byrne et al., 2023; Feleke et al., 2019; Jeang et al., 2023; Khaireh et

			al., 2012; Koffi et al., 2017; Labadie-Bracho, van Genderen, & Adhin, 2020; Leonard et al., 2022; Macalinao et al., 2023; Perraut et al., 2017; Rogier et al., 2018; Rogier et al., 2017)
	P. falciparum	PfGLURP-Ro	(Arzika et al., 2022; Koffi et al., 2017; Labadie-Bracho, van Genderen, & Adhin, 2020; Leonard et al., 2022; Lu et al., 2020; Perraut et al., 2017; Rogier et al., 2018)
		PfGLURP-R2	(Byrne et al., 2023; Kerkhof et al., 2016; Macalinao et al., 2023) remove kerhof
		PfHRP2	(Arzika et al., 2022; Rogier et al., 2018)
		PfSALSA	(Koffi et al., 2017; Perraut et al., 2017)
		PfPf13	(Koffi et al., 2017; Perraut et al., 2017)
		PfSEA1	(Byrne et al., 2023; Leonard et al., 2022; Macalinao et al., 2023)
		(Pf)Etramp5.Ag1	(Byrne et al., 2022; Byrne et al., 2023; Leonard et al., 2022; Macalinao et al., 2023)
		(Pf)GEXP18	(Byrne et al., 2023; Macalinao et al., 2023)
		(Pf)MSP2 CH150	(Byrne et al., 2023; Macalinao et al., 2023)
		(Pf)MSP2 Dd2	(Byrne et al., 2023; Macalinao et al., 2023)
		(Pf)EBA175 RII-V	(Jeang et al., 2023)
		PfMSP3	(Jeang et al., 2023)
		PfRH2ab	(Jeang et al., 2023)
	P. vivax	PvMSP1-19	(Arzika et al., 2022; Assefa et al., 2019; Byrne et al., 2023; Feleke et al., 2019; Herman et al., 2023; Jeang et al., 2023; Kerkhof et al., 2016; Khaireh et al., 2012; Labadie-Bracho, van Genderen, & Adhin, 2020; Leonard et al., 2022; Lu et al., 2020; Macalinao et al., 2023; McCaffery et al., 2022; Monteiro et al., 2021; Oviedo et al., 2022; Plucinski et al., 2018; Rogier et al., 2018; Rogier et al., 2017) remove kerhof
		PvMSP1-42	(Khaireh et al., 2012)
		chPvMSP1	(Leonard et al., 2022)
		PvRMC-MSP1	(McCaffery et al., 2022)

		PvAMA1	(Assefa et al., 2019; Byrne et al., 2022; Kerkhof et al., 2016; Leonard et al., 2022; Macalinao et al., 2023) remove kerhof
	P. vivax	PvEBP	(Byrne et al., 2023; Macalinao et al., 2023)
		PvR11	(Macalinao et al., 2023)
		PvDBP R11	(Byrne et al., 2023; Macalinao et al., 2023)
		PvRBD 1a	(Macalinao et al., 2023)
		PvDBPII (Sal1)	(Jeang et al., 2023)
		PvEBP2	(Jeang et al., 2023)
		PvRBP2b	(Jeang et al., 2023)
		PvDBPII	(Byrne et al., 2023)
		PvEBPII	(Byrne et al., 2022)
	P. ovale	PoMSP1-19	(Arzika et al., 2022; Feleke et al., 2019; Herman et al., 2023; Plucinski et al., 2018)
	P. malariae	PmMSP1-19	(Arzika et al., 2022; Assefa et al., 2019; Byrne et al., 2022; Feleke et al., 2019; Herman et al., 2023; Labadie-Bracho, van Genderen, & Adhin, 2020; Lu et al., 2020; Monteiro et al., 2021; Njenga et al., 2020; Oviedo et al., 2022; Plucinski et al., 2018; Rogier et al., 2018; van den Hoogen et al., 2021)
		PmCSP	(Koffi et al., 2017; Perraut et al., 2017)
		PmMSP1-F2	(Monteiro et al., 2021)
		PmMSP1-F2	(Monteiro et al., 2021)
		PmMSP1-F3	(Monteiro et al., 2021)
		PmMSP1-F4	(Monteiro et al., 2021)
	Non-specific peptides	Lsa1-41, Lsa1-J, Lsa3-NR2, Glurp, GlurpP3, Salsa1, Salsa2, Trap1, Starp-R, CS (NANP) band SR11	(Khaireh et al., 2012)
Lymphatic Filariasis	W. bancrofti	Wb123	(Cadavid Restrepo et al., 2022; Y. Chan et al., 2022; Y. L. Chan et al., 2022; Fornace et al., 2022; Njenga et al., 2020; Plucinski et al., 2018; Priest et al., 2016; Won et al., 2018)

	B. malayi	Bm14	(Cadavid Restrepo et al., 2022; Y. Chan et al., 2022; Y. L. Chan et al., 2022; Moss et al., 2011; Njenga et al., 2020; Plucinski et al., 2018; Priest et al., 2016; Won et al., 2018)
	B. malayi	Bm33	(Cadavid Restrepo et al., 2022; Y. Chan et al., 2022; Y. L. Chan et al., 2022; Moss et al., 2011; Njenga et al., 2020; Plucinski et al., 2018; Priest et al., 2016; Won et al., 2018)
		Soluble lysates	(Kwan et al., 2018)
		SXP1	(Fujii et al., 2014)
		BmR1	(Y. L. Chan et al., 2022)
Strongyloidiasis	S. stercoralis	NIE	(Y. Chan et al., 2022; Y. L. Chan et al., 2022; Fornace et al., 2022; Njenga et al., 2020; Plucinski et al., 2018; Priest et al., 2016)
		Soluble lysates	(Kwan et al., 2018)
Dengue	Dengue virus	DENV-1	
		DENV-2	(Y. Chan et al., 2022; Poirier et al., 2016)
		DENV-3	(Poirier et al., 2016)
		DENV-4	
Chikungunya	Chikungunya	E1	(Y. Chan et al., 2022; Poirier et al., 2016)
Schistosomiasis	S. masoni	GST	(Njenga et al., 2020)
		SEA	(Fornace et al., 2022; Njenga et al., 2020)
		Sm25	(Njenga et al., 2020)
Trachoma	C. trachomatis	Pgp3	(Aiemjoy et al., 2020; Y. Chan et al., 2022; Y. L. Chan et al., 2022; Cooley et al., 2021; Fornace et al., 2022; Mentzer et al., 2022)
		Ct694	(Aiemjoy et al., 2020; Y. Chan et al., 2022; Y. L. Chan et al., 2022; Cooley et al., 2021)
Yaws	T. pallidum	Rp17	(Y. Chan et al., 2022; Y. L. Chan et al., 2022; Cooley et al., 2021; Fornace et al., 2022)
		TmpA	(Y. Chan et al., 2022; Y. L. Chan et al., 2022; Cooley et al., 2021; Fornace et al., 2022)
Onchocerciasis	O. volvulus	Ov16	(Fornace et al., 2022)

Leishmaniasis	L. donovani	KRP42	(Fujii et al., 2014)
Cysticercosis	Cysticercosis	T24H	(Priest et al., 2016)
		Hydatid cyst fluid?	(Miernyk et al., 2019)
Diphtheria	Diphtheria	Toxoid	(Boey et al., 2021; Breakwell et al., 2020; Caboré, Piérard, & Huygen, 2016; Khetsuriani et al., 2022; Minta et al., 2020; Njenga et al., 2020; Tohme et al., 2023)
Tetanus	Tetanus	Toxoid	(Boey et al., 2021; Breakwell et al., 2020; Caboré, Piérard, & Huygen, 2016; Khetsuriani et al., 2022; Minta et al., 2020; Njenga et al., 2020; Tohme et al., 2023)
Pertussis	Pertussis	Toxoid	(Boey et al., 2021; Caboré, Piérard, & Huygen, 2016)
		FHA	(Boey et al., 2021; Caboré, Piérard, & Huygen, 2016)
		Prn	(Boey et al., 2021; Caboré, Piérard, & Huygen, 2016)
Rubella	Rubella	Inactivated rubella	(Breakwell et al., 2020; Minta et al., 2020)
Measles	Measles	MV-n	(Breakwell et al., 2020; Minta et al., 2020; Njenga et al., 2020)
Varicella	Varicella	Varicella	(Mentzer et al., 2022)
Giardia	G. lamblia	VSP1	(Mosites et al., 2018)
		VSP2	(Mosites et al., 2018)
		VSP3	(Aiemjoy et al., 2020; Arnold et al., 2019; Arzika et al., 2022; Y. L. Chan et al., 2022; Fornace et al., 2022; Miernyk et al., 2019; Mosites et al., 2018; Zambrano et al., 2017)
		VSP4	(Mosites et al., 2018)
		VSP5	(Aiemjoy et al., 2020; Arnold et al., 2019; Arzika et al., 2022; Y. L. Chan et al., 2022; Miernyk et al., 2019; Mosites et al., 2018; Zambrano et al., 2017)
Bacterial intestinal infections	enterotoxigenic E. Coli	ETEC-LTB	(Aiemjoy et al., 2020; Arnold et al., 2019; Arzika et al., 2022; Y. Chan et al., 2022; Zambrano et al., 2017) not Zambrano 2017 here
		ETEC-Etxb	(Zambrano et al., 2017)
	Salmonella species	Salmonella LPS	(Arnold et al., 2019; Arzika et al., 2022)
		Salmonella LPS Group B	(Aiemjoy et al., 2020; Arnold et al., 2019)
		Salmonella LPS Group D	(Aiemjoy et al., 2020; Arnold et al., 2019)

	V. cholera	CtxB	(Aiemjoy et al., 2020; Arnold et al., 2019; Zambrano et al., 2017)
		CTX	(Fujii et al., 2014)
	H. pylori		(Mentzer et al., 2022)
Other bacterial	Campylobacter	P18	(Aiemjoy et al., 2020; Arnold et al., 2019; Arzika et al., 2022; Zambrano et al., 2017)
		P38	(Arzika et al., 2022)
		P39	(Aiemjoy et al., 2020; Arnold et al., 2019; Zambrano et al., 2017)
	Group A strep	SPEB	(Arzika et al., 2022)
Parasitic intestinal infections	E. histolytica	LecA	(Aiemjoy et al., 2020; Arnold et al., 2019; Y. Chan et al., 2022; Zambrano et al., 2017)
		C-lgL	(Fujii et al., 2014)
	T. gondii	SAGA2	(Y. Chan et al., 2022; Y. L. Chan et al., 2022; Priest et al., 2016)
		Sag1	(Fujii et al., 2014; Mentzer et al., 2022)
		P22	(Mentzer et al., 2022)
Cryptosporidium	C. parvum	Cp23	(Aiemjoy et al., 2020; Arnold et al., 2019; Arzika et al., 2022; Y. Chan et al., 2022; Miernyk et al., 2019; Mosites et al., 2018; Zambrano et al., 2017)
		Cp17	(Aiemjoy et al., 2020; Arnold et al., 2019; Arzika et al., 2022; Miernyk et al., 2019; Mosites et al., 2018; Zambrano et al., 2017)
Respiratory viruses	SARS-CoV-2	RBD	(Woudenberg et al., 2021)
		Spike S2	(Woudenberg et al., 2021)
		Nucleocapsid	(Woudenberg et al., 2021)
		Membrane-envelope	(Woudenberg et al., 2021)
	Human coronaviruses	229E	(Woudenberg et al., 2021)
		HKU1	(Woudenberg et al., 2021)
		NL63	(Woudenberg et al., 2021)
		OC43	(Woudenberg et al., 2021)
	Norovirus	Norwalk VLP	(Zambrano et al., 2017)

		Sydney VLP	(Zambrano et al., 2017)
		St Cloud VLP	(Zambrano et al., 2017)
		GI.4	(Arnold et al., 2019)
		GII.NO	(Arnold et al., 2019)
Herpes virus family	Epstein Barr	EBV VCAp18<br>EBV EBNA1<br>EBV ZEBRA<br>EBV EA-D	(Mentzer et al., 2022)
	Herpes Simplex	HSV-1<br>HSV-2<br>VZV	(Mentzer et al., 2022)
	Cytomegalovirus	CMV pp150N<br>CMV pp52<br>CMV pp28	(Mentzer et al., 2022)
	Kaposi's sarcoma-associated herpesvirus	KSHV LANA<br>KSHV K8.1	(Mentzer et al., 2022)
	Human Herpes	HHV-6 IE1A<br>HHV-6 IE1B<br>HHV-6 p101k<br>HHV-7 U14	(Mentzer et al., 2022)
Other viruses	Hepatitis B and C	HBV Core<br>HBV NS3<br>HCV Core<br>HCV NS3	(Mentzer et al., 2022)
	HIV	Gag	(Fujii et al., 2014; Mentzer et al., 2022)
		env	(Mentzer et al., 2022)
		Gp41	(Fujii et al., 2014)
		Gp120	(Fujii et al., 2014)
	Human T-lymphotropic	HTLV-1 Gag<br>HTLV-1 Env	(Mentzer et al., 2022)
	Human Papilloma	HPV-16 L1<br>HPV-18 L1	(Mentzer et al., 2022)
	John Cunningham	JCV VP1<br>BKV VP	(Mentzer et al., 2022)

	BK Virus		(Mentzer et al., 2022)
	Hb-ascaris suum		(Njenga et al., 2020)

S4: Seropositivity determination method and prevalence findings

Study ID	Seropositivity determination	Prevalence findings (overall conclusion)
Aiemjoy 2020	ROC; Presumed unexposed (usually mean+/- 3SD); Mixture model	C. trachomatis - 43.1% (95% CI: 38 - 48.4)<br>S. enterica - 27.5% (95% CI: 23.6 - 31.6)<br>E. histolytica - 70.3% (95% CI: 67.7 - 72.8)<br>G. intestinalis - 53.9% (95% CI: 51.8 - 56.0)<br>C. jejuni - 95.6% (95% CI: 94.4 - 96.5)<br>ETEC - 76.3% (95% CI: 74.1 - 78.4)<br>C. parvum - (95% CI: 92.8 - 94.9)
Arnold 2019	ROC; Presumed unexposed (usually mean+/- 3SD); Mixture model	Seropositivity defined by crossing seropositivity cut-offs and measured in incident cases per child-years (Rate, 95% CI)<br>Seroconversion/boosting:<br>Giardia VSP-3 or VSP-5: 0.40 (95% CI: 0.34 - 0.48)<br>Cryptosporidium Cp17 or Cp23: 0.64 (95% CI: 0.54 - 0.77)<br>E. hisolytica LecA: 0.34 (95% CI: 0.28 - 0.42)<br>Salmonella LPS Groups B or D: 0.57 (95% CI: 0.47 - 0.68)<br>ETEC LT B subunit: 1.13 (95% CI: 0.75 - 1.82)<br>Norovirus GI.4: 0.38 (95% CI: 0.30 - 0.47)<br>Norovirus GII.4.NO: 0.63 (95% CI: 0.51 - 0.80)<br>Seroreversion/waning:<br>Giardia VSP-3 or VSP-5: 0.21 (95% CI: 0.17 - 0.25)<br>Cryptosporidium Cp17 or Cp23: 0.05 (95% CI: 0.03 - 0.07)<br>E. histolytica LecA: 0.11 (95% CI: 0.08 - 0.15)<br>Salmonella LPS group B or D: 0.05 (95% CI: 0.03 - 0.07)<br>ETEC LT B subunit: 0.00 (95% CI: 0.00 - 0.01)<br>Norovirus GI.4: 0.06 (95% CI: 0.03 - 0.09)<br>Norovirus GI.4.NO: 0.03 (95% CI: 0.02 - 0.05)
Arzika 2022	ROC; Presumed unexposed (usually mean+/- 3SD)	Baseline seroprevalence:<br>Placebo group:<br>PfMSP1-19: 79%<br>PfAMA1: 67%<br>PfGLURP-Ro: 26%<br>PfLSA1: 11%<br>PfCSP: 5%<br>PfHRP2: 2%<br>PmMSP1-19: 13%<br>PoMSP1-19: 2%<br>PvMSP1-19: 1%<br>Campylobacter p18 or p39: 92%<br>ETEC LTB: 88%<br>Salmoella LPS: 48%<br>Cryptosporidium cp17 or cp23: 85%<br>Giardia VSP3 or VSP5: 77%<br>Strep A SPEB: 63%<br>Azithromycin group:<br>PfMSP1-19: 76%<br>PfAMA1: 61%<br>PfGLURP-Ro: 21%<br>PfLSA1: 8%<br>PfCSP: 4%<br>PfHRP2: 2%

		PmMSP1-19: 12%<br>PoMSP1-19: 3%<br>PvMSP1-19: 2%<br>Campylobacter p18 or p39: 91%<br>ETEC LTB: 84%<br>Salmoella LPS: 45%<br>Cryptosporidium cp17 or cp23: 83%<br>Giardia VSP3 or VSP5: 76%<br>Strep A SPEB: 60%
Assefa 2019	Mixture model	All ages seropositivity:<br>PfMSP-1 - 23.8% (95% CI: 21.4 - 26.3)<br>PfAMA-1 - 24.5% (95% CI: 22.4 - 26.7)<br>PvMSP-1 - 21.2% (95% CI: 19.2 - 23.4)<br>PvAMA-1 - 14.4% (95% CI: 12.7 - 15.2)<br>PmMSP-1 - 8.6% (95% CI: 7.6 - 9.7)<br>PoMSP-1 - 3.1% (95% CI: 2.5 - 3.8)
Boey 2021	International Standard units	Seroprotection (overall):<br>Diphtheria (>0.1 IU/mL): 28.9% (95% CI: 26.2 - 31.7)<br>Tetanus (>0.1 IU/mL): 82.6% (95% CI: 80.2 - 84.8)<br>Seronegative (overall):<br>Diphtheria (<0.01 IU/mL): 35.6% (95% CI: 32.7 - 38.6)<br>Tetanus (<0.01 IU/mL): 2.4% (95% CI: 1.5 - 3.5)
Breakwell 2020	International Standard units	Minimal protective immunity overall:<br>Measles 99% (95% CI: 98-99)<br>Rubella 99% (95% CI: 97 - 100)<br>Tetanus 85% (95% CI: 83 - 87)<br>Diphtheria 51% (95% CI: 47 - 55)
Byrne 2022	Presumed unexposed (usually mean+/-3SD); Other	Paktha (n=983) P. vivax historic 308 (0.31%) and recent 88 (0.09%)<br>Paktha P. falciparum historic 52 (0.05%) and recent 21 (0.02%)<br>Khua/Nambak (n=2,393) P. vivax historic 470 (0.2%) and recent 46 (0.02%)<br>Khua/Nambak P. falciparum historic 46 (0.02%) and recent 24 (0.01%)<br>Et (n=1,418) P. vivax historic 289 (0.2%) and recent 52 (0.04%)<br>Et P. falciparum 19 historic (0.01%) and recent 10 (0.0%)
Byrne 2023	Mixture model; Pre-exposed endemic cohort	NIL positive identification from microscopy;<br>55 (0.005%) positive for P. falciparum by PCR;<br>0.04% (95% CI: 0.036-0.044) Recent exposure to P. falciparum<br>32.4% (95% CI: 31.4-33.4%) historical exposure to P. falciparum<br>16.4% (95% CU: 15.6-17.1) historical exposure to p. vivax
Cabora 2016	International Standard units	Antibody concentration below protective level:<br>Diphtheria: 26.4%<br>Tetanus: 8.6%<br>Seroprotection:<br>Diphtheria: 73.6%<br>Tetanus: 90.7%<br>Pertussis infection during last few years: 6.8%
CadavidRestrepo 2022	Presumed unexposed	TAS1:<br>Bm33 - 12% (95% CI: 10.1 - 14)

	(usually mean+/-3SD)	Bm14 - 6.8% (95% CI: 5.4 - 8.5)<br>Wb123 - 1.0% (95% CI: 0.5 - 17.6)<br>TAS2:<br>Bm33 - 7.8% (95% CI: 10.1 - 14)<br>Bm14 - 3% (95% CI: 2 - 4.4)<br>Wb123 - 3.6% (95% CI: 2.4 - 5.1)<br>TAS3:<br>Bm33 - 20.8% (95% CI: 18.5 - 23.3)<br>Bm14 - 1.6% (95% CI: 0.9 - 2.5)<br>Wb123 - 8.3% (95% CI: 6.7 - 10)
Chan 2022	Mixture model; Pre-exposed endemic cohort	Bm33 - 10.9%<br>Wb123 - 1.72%<br>Bm14 - 3.56%<br>BmR1 - 2.46%<br>Ss NIE (<3 years) - 16.8%<br>Sag2A - 29.9%<br>Rp17 (<14 years) - 4.91%<br>TmpA (<14 years) - 4.87%<br>Rp17 + TmpA (double seropositive) - 1.16%<br>VSP3 (<5 years) MFI > 38.37%<br>VSP5 (<5 years) MFI > 29.21%<br>VSP3 + VSP5 (double seropositive) - 23.24% Trachmoa 23.24%
Chan 2022	ROC; Presumed unexposed (usually mean+/-3SD); Mixture model	PfMSP1-19: 21.8%<br>Sag2A: 45.0%<br>Wb123: 1.3%<br>Bm14: 2.8%<br>Bm33: 7.2%<br>NIE: 9.2%<br>Chik E1: 43.5%<br>Dengue 2 VLP: 75.6%<br>Pgp3: 41.7%<br>Ct694: 35.2%<br>rp17: 6.6%<br>TmpA: 5.0%<br>LecA: 7.2%<br>Cp23: 26.1%
Cooley 2021	ROC	Nayapara:<br>Pgp3: 1.5% (95% CI: 0.47 - 4.0)<br>Ct694: 7.0% (95% CI: 4.4 - 10.8)<br>Rp17: 2.4% (95% CI: 0 - 0.5)<br>TmpA: 0.37% (95% CI: 0 - 2.3)<br>Both antigens Rp17 and TmpA: 0% (95% CI: 0 - 1.7)<br>Makeshift settlements (1-9 years):<br>Pgp3: 1.4% (95% CI: 0.67 - 2.8)<br>Ct694: 2.8% (95% CI: 1.7 - 4.6)<br>Makeshift settlements (1-14 years):<br>Rp17: 3.3% (95% CI: 2.1 - 5.0)<br>TmpA: 0.91% (95% CI: 0.3 - 2.0)<br>Both antigens Rp17 and TmpA: 0.15% (95% CI: 0 - 0.18)<br>Makeshift settlements (all):<br>Rp17: 2.9% (95% CI: 0.9 - 4.9)<br>TmpA: 0.73% (95% CI: 0 - 1.7)<br>Both antigens Rp17 and TmpA: 0% (95% CI: 0 - 1.1)

Feldstein 2020	ROC;<br>International Standard units	Makeshift settlements for ages 1-6 years:<br>Measles - 91% (95% CI: 86 - 94)<br>Rubella - 84% (95% CI: 79 - 88)<br>Diphtheria - 63% (95% CI: 56 - 70)<br>Tetanus - 76% (95% CI: 69 - 81)<br>Makeshift settlements for ages 7-14 years:<br>Measles - 99% (95% CI: 96 - 100)<br>Rubella - 96% (95% CI: 90 - 98)<br>Diphtheria - 77% (95% CI: 69 - 84)<br>Tetanus - 84% (95% CI: 77 - 89)<br>Nayapara ages 1-6 years:<br>Measles 97% (95% CI: 94 - 99)<br>Rubella - 98% (5% CI: 95 - 99)<br>Diphtheria - 91% (95% CI: 87 - 94)<br>Tetanus - 97% (95% CI: 95 - 99)
Feleke 2019	Presumed unexposed (usually mean+/-3SD)	PvMSP1-19: 28%<br>PfAMA1: 33%<br>PfMSP1-19: 29%<br>PfCSP: 9%<br>PfLSA1: 2%<br>PmMSP1-19: 11%<br>PoMSP1-19: 7%
Fornace 2022	Mixture model	Pgp3: 1.9%<br>Wb123: 0.5%<br>Ov16: 1.1%<br>NIE: 2.6%<br>SEA: 13.5%<br>rp17: 0.2%<br>TmpA: 0.1%<br>VSP3: 8.9%
Fujii 2014	Presumed unexposed (usually mean+/-3SD)	Kwale site:<br>HIV - 3% (95% CI: 2.1 - 3.9)<br>W. bancrofti - 21.8% (95% CI: 19.7 - 23.9)<br>L. donovani - 12.6% (95% CI: 10.9 - 14.3)<br>E. histolytica - 12.8% (95% CI: 11.1 - 14.5)<br>V. cholerae - 31.7% (95% CI: 29.3 - 34.1)<br>T. gondii - 30.9% (95% CI: 28.5 - 33.3)<br>Mbita site:<br>HIV - 20.1% (95% CI: 18.3 - 21.8)<br>W. bancrofti - 13.5% (95% CI: 12.0 - 15.0)<br>L. donovani - 17.3% (95% CI: 15.6 - 19.0)<br>E. histolytica - 16.6% (95% CI: 15 - 18.2)<br>V. cholerae - 24.9% (95% CI: 23 - 26.8)<br>T. gondii - 28.2% (95% CI: 26.2 - 30.2)
Herman 2023	Mixture model	PfMSP1: "A report outlining comprehensive analyses for P. falciparum infection and exposure among persons of all ages from NAIIS 2018 is forthcoming"<br>PvMSP1: 6.3% (95% CI: 6.0 - 6.7)<br>PoMSP1: 12.1% (95% CI: 11.6 - 12.5)<br>PmMSP1: 34.2% (95% CI: 33.3 - 35.2)
Jeang 2023	Presumed unexposed (usually mean+/-3SD)	Arjo region (n=473)<br>PfAMA1: 65.12%<br>PfCSP: 10.99%<br>PfEBA175RII-V: 49.89%

		PfMSP1-42: 64.90%<br>PfMSP3: 23.68%<br>PfRH2ab: 36.58%<br>PvDBPII(Sal1): 38.69%<br>PvEBP2: 40.17%<br>PvMSP1-19: 57.72%<br>PvRBP2b: 45.67%<br>Gambella region (n=328)<br>PfAMA1: 96.04%<br>PfCSP: 38.41%<br>PfEBA175RII-V: 86.89%<br>PfMSP1-42: 97.26%<br>PfMSP3: 70.12%<br>PfRH2ab: 76.83%<br>PvDBPII(Sal1): 64.02%<br>PvEBP2: 67.68%<br>PvMSP1-19: 89.63%<br>PvRBP2b: 61.28%
Khaireh 2012	Presumed unexposed (usually mean+/-3SD)	P. falciparum - 31.5% (95% CI: 29.4 - 33.7)<br>P. vivax - 17.5% (95% CI: 15.8 - 19.3)<br>Mixed infection - 10.2% (95% CI: 8.9 - 11.7)
Khetsuriani 2022	International Standard units	Adjusted Diphtheria and tetanus antibody levels by survey site:<br>Zakarpattya (n=1,306)<br>Diphtheria:<br>No protection - 18.7% (95% CI: 13 - 26.1)<br>Minimal protection - 31.3 (95% CI: 27.7 - 35.2)<br>Protection - 33.8% (95% CI: 28.4 - 39.6)<br>Full protection - 16.2% (95% CI: 12.7 - 20.5)<br>Tetanus:<br>No protection - 21.5% (95% CI: 14.6 - 30.6)<br>Minimal protection - 16.9 (95% CI: 13.7 - 20.6)<br>Protection 27.1% (95% CI: 22.9 - 31.7)<br>Full protection - 34.5% (95% CI: 28 - 41.7)<br>Sumy (n=1,403)<br>Diphtheria<br>No protection - 4.4% (95% CI: 3.2 - 6.2)<br>Minimal protection - 19.3% (95% CI: 16.3 - 22.7)<br>Protection - 44.5% (95% CI: 41.4 - 47.8)<br>Full protection - 31.8% (95% CI: 28.6 - 35.2)<br>Tetanus:<br>No protection - 4.4% (95% CI: 3.3 - 5.8)<br>Minimal protection - 6.5% (95% CI: 4.6 - 9)<br>Protection - 26.6% (95% CI: 23.4 - 30.1)<br>Full protection - 62.5% (95% CI: 57.4 - 67.4)<br>Odessa (n=1,298)<br>Diphtheria:<br>No protection - 7.9% (95% CI: 5.8 - 10.8)<br>Minimal protection - 22.8% (95% CI: 19.8 - 26.1)<br>Protection - 44.2% (95% CI: 40.7 - 47.9)<br>Full protection - 25.1% (95% CI: 21.9 - 28.4)<br>Tetanus:<br>No protection - 7.9% (95% CI: 5.5 - 11.1)<br>Minimal protection - 11.5% (95% CI: 9.2 - 14.2)

		Protection - 32.9% (95% CI: 29.1 - 36.9)<br>Full protection - (95% CI: 47.8% (95% CI: 43.1 - 52.5)<br>Kyiv City (n=722)<br>Diphtheria:<br>No protection - 3.5% (95% CI: 2.3 - 5.2)<br>Minimal protection - 17.3% (95% CI: 15.3 - 19.5)<br>Protection - 49.2% (95% CI: 43.7 - 54.7)<br>Full protection - 30% (95% CI: 27.1 - 33.1)<br>Tetanus:<br>No protection - 4.3% (95% CI: 3 - 6.2)<br>Minimal protection - 6.8% (95% CI: 4.8 - 9.5)<br>Protection - 32.4% (95% CI: 27.3 - 38)<br>Full protection - 56.4 (95% CI: 51.1 - 61.6)
Koffi 2017	Presumed unexposed (usually mean+/- 3SD)	Listed in order of year (2010-2011(RDT + in 2011)-2011 (RDT - in 2011)-2012-2013)<br>CSP: 40.4% (2010); 20.0% (2011 - RDT+ in 2011); 33.3% (2011 RDT - in 2011); 32.1% (2012); 41.9% (2013)<br>LSA1-41: 64.9%; 25.7%; 41.7%; 48.2%; 53.2%<br>LSA3: 12.3%; 8.6%; 4.2%; 5.4%; 6.5%<br>SALSA: 80.7%; 88.6%; 70.8%; 76.8%; 71.0%<br>GLURP: 49.1%; 80.0%; 62.5%; 57.1%; 66.1%<br>AMA1: 56.1%; 57.1%; 41.7%; 50.0%; 40.3%<br>Pf13: 93.0%; 94.3%; 83.3%; 89.3%; 82.3%<br>MSP1p19: 93.0%; 88.6%; 100%; 94.6%; 96.8%<br>MSP4p20: 93.0%; 97.1%; 91.7%; 91.1%; 93.5%<br>PmCSP: 7.0%; 25.7%; 29.2%; 8.9%; 8.1%<br>gSG6: 29.8%; 2.9%; 8.3%; 8.9%; 6.5%
Kwan 2018	ROC	Age 6 months:<br>Filaria positive: 16.8%<br>Strongyloides positive: 8.1%<br>Age 12 months:<br>Filaria positive: 18.9%<br>Strongyloides positive: 3.1%<br>Age 1.5 years:<br>Filaria positive: 32.9%<br>Strongyloides positive: 4.5%<br>Age 2 years:<br>Filaria positive: 39.2%<br>Strongyloides positive: 5.4%<br>Age 2.5 years:<br>Filaria positive: 60.0%<br>Strongyloides positive: 8.0%
Labadie-Bracho 2020	Presumed unexposed (usually mean+/- 3SD)	Note: Surinamese participants from Stoelmanseiland area (n=86)<br>Note: Brazilian participants from Benzdorp region (n=65)<br>AMA-1 (Surinamese participants) - 72.1%<br>AMA-1 (Brazilian participants) - 52.3%<br>PfMSP1-19 (Surinamese participants) - 66.3%<br>PfMSP1-19 (Brazilian participants) - 60.0%<br>GLURP (Surinamese participants) - 62.8%<br>GLURP (Brazilian participants) - 41.5%<br>CSP (Surinamese participants) - 22.1%<br>CSP (Brazilian participants) - 24.6%

		LSA (Surinamese participants) - 38.4%<br>LSA (Brazilian participants) - 16.9%
Leonard 2022	Presumed unexposed (usually mean+/-3SD)	Bead antigen positive (includes positive result for any malaria antigen) - 1.8% (95% CI: 1.2 - 2.3)<br>Short term Pf positive - 30.8% (95% CI: 28.9 - 32.7)<br>Long term Pf positive - 38.1% (95% CI: 36.1 - 40.1)<br>Pv positive - 39.9% (95% CI: 37.9 - 41.9)<br>Any IgG positive - 58.2% (95% CI: 56.2 - 60.3)
Lu 2020	Presumed unexposed (usually mean+/-3SD)	Nayapara:<br>PmMSP1: 0.7%<br>PvMSP1: 1.8%<br>PfMSP1: 1.1%<br>Pf short term: 0.7%<br>Any plasmodium: 4.0%<br>Kutupalong:<br>PmMSP1: 2.3%<br>PvMSP1: 3.9%<br>PfMSP1: 1.3%<br>Pf short term: 0.3%<br>Any plasmodium: 7.1%<br>Makeshift:<br>PmMSP1: 2.0%<br>PvMSP1: 3.4%<br>PfMSP1: 2.9%<br>Pf short term: 1.5%<br>Any plasmodium: 8.7%
Macalinao 2023	Presumed unexposed (usually mean+/-3SD); Mixture model	Palawan (n=6572)<br>PfAMA1: 58.5%<br>PfMSP1-19: 58.9%<br>Etramp5.Ag1: 40.5%<br>GEXP18: 38.4%<br>PfGLURP-R2: 58.1%<br>MSP2-CH150/9: 47.1%<br>MSP2Dd2: 55.9%<br>PfSEA: 36.3%<br>PvAMA1: 45.5%<br>PvMSP1-19: 39.4%<br>PvDBP.RII: 25.9%<br>PvRBP-1a: 30.1%<br>PvRII: 16.4%<br>PvEBP: 50.6%<br>Occidental Mindoro (n=1683)<br>PfAMA1: 35.6%<br>PfMSP1-19: 38.0%<br>Etramp5.Ag1: 15.9%<br>GEXP18: 12.1%<br>PfGLURP-R2: 44.4%<br>MSP2-CH150/9: 24.6%<br>MSP2Dd2: 39.0%<br>PfSEA: 27.9%<br>PvAMA1: 38.6%<br>PvMSP1-19: 25.5%<br>PvDBP.RII: 9.8%<br>PvRBP-1a: 24.7%

		PvRII: 6.2%<br>PvEBP: 42.7%<br>Bataan (n=877)<br>PfAMA1: 10.4%<br>PfMSP1-19: 9.7%<br>Etramp5.Ag1: 6.8%<br>GEXP18: 7.4%<br>PfGLURP-R2: 16.8%<br>MSP2-CH150/9: 8.7%<br>MSP2Dd2: 14.4%<br>PfSEA: 14.9%<br>PvAMA1: 9.6%<br>PvMSP1-19: 7.2%<br>PvDBP.RII: 5.7%<br>PvRBP-1a: 27.6%<br>PvRII: 4.3%<br>PvEBP: 12.1%
McCaffery 2022	Presumed unexposed (usually mean+/-3SD)	Ethiopia PfMSP1 = 27.6%<br>Ethiopia PvMSP1 = 21.4% and PvRMC-MSP1 = 32.6%<br>Costa rica PfMSP1 = 3.6%<br>Costa rica PvMSP1 = 41.5% and PvRMC-MSP1 = 46.7%
Mentzer 2022	Other: Calculations from antigen reactivity data	Overall prevalence:<br>HSV-1 - 69.8% (95% CI: 68.9 - 70.7)<br>HSV-2 - 16.2% (95% CI: 15.5 - 16.9)<br>VZV - 92.5% (95% CI: 92 - 93)<br>EBV - 94.7% (95% CI: 94.3 - 95.2)<br>CMV - 58.2% (95% CI: 57.2 - 59.2)<br>HHV-6A or 6B - 90.8% (95% CI: 90.2 - 91.4)<br>HHV-7 - 94.7% (95% CI: 94.3 - 95.2)<br>KSHV - 8.1% (95% CI: 7.5 - 8.6)<br>HBV - 2.5% (95% CI: 2.2 - 2.8)<br>HCV - 0.3% (95% CI: 2.2 - 2.8)<br>HIV-1 - 0.2% (95% CI: 0.1 - 0.3)<br>HTLV-1 - 1.6% (95% CI: 1.3 - 1.8)<br>HPV-16 - 4.4 (95% CI: 4.2 - 4.8)<br>HPV-18 2.8% (95% CI: 2.4 - 3.1)<br>JCV - 57.5 (95% CI: 56.5 - 58.5)<br>BKV - 95.4% (95% CI: 95 - 95.8)<br>T. gondii - 28% (95% CI: 27.1 - 28.9)<br>C. trachomatis - 21.4% (95% CI: 20.6 - 22.2)<br>H. pylori - 35.3% (95% CI: 34 - 36.6)
Miernyk 2019	Other: Double positivity for Cryptosporidium and G. intestinalis	Unadjusted seropositivity:<br>Cryptosporidium spp - 28.8%<br>Bunyavirus - 26.6%<br>Giardia intestinalis - 18.9%<br>Coxiella burnetii - 8.3%<br>Trichinella spp - 5.0%<br>Toxoplasma gondii - 2.9%<br>Hep E - 2.6%<br>E. granulosus - 1.8%<br>E. multiocularis - 0.1%<br>Brucella spp - 0.1%<br>F. tularensis - 0.0%<br>>1 pathogen - 62.6%

Minta 2020	International Standard units	Tetanus 83% (95% CI: 80-86%)<br>Diphtheria 83% (95% CI: 81-84%)<br>Measles 87% (95% CI: 85-89%)<br>Rubella 84% (95% CI: 81-87%)
Monteiro 2021	Presumed unexposed (usually mean+/-3SD)	P. vivax - 52.8%<br>P. falciparum - 21.5%
Mosites 2018	Presumed unexposed (usually mean+/-3SD)	Giardia - 18.94%<br>Cryptosporidium - 28.86%
Moss 2011	Presumed unexposed (usually mean+/-3SD)	Pre-treatment:<br>Bm14 IgG - 79.05%<br>Bm33 IgG - 92.56%<br>Bm14 IgG4 - 45.94%<br>Bm33 IgG4 - 39.18%<br>PfPTP3 IgG - 12%<br>PfMSP1-19 IgG - 19%<br>6-months post treatment<br>Bm14 IgG - 78.37%<br>Bm33 IgG - 91.89%<br>Bm14 IgG4 - 47.29%<br>Bm33 IgG4 - 29.05%<br>PfPTP3 IgG - 16%<br>PfMSP1-19 IgG - 18%
Njenga 2020	ROC; International Standard units; Presumed unexposed (usually mean+/-3SD); Mixture model	Antibody responses to the P. falciparum CSP and MSP-119 antigens increased with age in communities in Kilifi and Kwale counties, with higher seroprevalence in Jaribuni community. Mean antibody responses against P. malariae MSP-119 antigen also increased with age and were highest in Jaribuni.<br>Antibody responses against S. mansoni Sm25 recombinant antigen were primarily detected in Kimorigo community in Taitaâ€“Taveta County, and the seroprevalence increased gradually with age, reaching a peak at around 25 years of age.<br>Generally, diphtheria seroprotection ranged between 22% and 44% across communities, and partial protection (defined as responses of 0.01â€“0.099 IU/mL) ranged between 70% and 88% across communities.
Oviedo 2022	Presumed unexposed (usually mean+/-3SD)	PmMSP1-19: 1.27%<br>PvMSP1-19: 0.46%<br>Both PmMSP1-19 and PvMSP1-19: 0.06%
Perraut 2017	Presumed unexposed (usually mean+/-3SD)	Prevalence (%) in 2002 and 2013 (% change)<br>Dielmo (2002 n=184; 2013 n=196)<br>Schizont extract - 93.4% - 64.9% (-30%)<br>PfCSP - 70.1% - 39.8% (-43%)<br>PfLSA1-4 - 84.2% - 62.2% (-26%)<br>PfSALSA - 53.8% - 29.1% (-46%)<br>PfGLURP - 78.3% - 50.5% (-35%)<br>PfAMA1 - 53.8% - 18.4% (-66%)<br>Pf13 - 87.5% - 44.9% (-49%)

		PfMSP1-p19 - 79.3% - 57.7% (-27%)<br>PmCSP - 79.3% - 49% (-38%)<br>Ndiop (2002 n=202; 2013 n=216)<br>Schizont extract - 78.7% - 56.3% (-29%)<br>PfCSP - 58.9% - 39.8% (-32%)<br>PfLSA1-4 - 72.8% - 52.3% (-28%)<br>PfSALSA - 51.9% - 30.6% (-41%)<br>PfGLURP - 67.3% - 48.6% (-28%)<br>PfAMA1 - 50.9% - 19.4% (-62%)<br>Pf13 - 64.4% - 43.1% (-33%)<br>PfMSP1-p19 - 74.3% - 52.3% (-30%)<br>PmCSP - 42.1% - 29.6% (-30%)
Plucinski 2018	Presumed unexposed (usually mean+/- 3SD)	For Macala-a-velha (2013 - 2014)<br>PfMSP1-19: 80% - 80% (no change)<br>PfCSP: 61% - 58% (-5% change)<br>PfLASA-1: 31% - 28% (-1-% change)<br>PvMSP1-19: 3% - 2% (-37% change)<br>PoMSP1-19: 41% - 35% (-14% change)<br>PmMSP1-19: 46% - 40% (-14% change)<br>Wb123: 50% - 43% (-15% change)<br>Bm14: 37% - 32% (-12% change)<br>Bm33: 67% - 61% (-10% change)<br>NIE: 58% - 56% (-4% change)<br>For Mecuburi (2013-2014)<br>PfMSP1-19: 81% - 80% (-2% change)<br>PfCSP: 71% - 71% (no change)<br>PfLASA-1: 33% - 38% (+15% change)<br>PvMSP1-19: 4% - 2% (-54% change)<br>PoMSP1-19: 43% - 42% (-2% change)<br>PmMSP1-19: 56% - 54% (-3% change)<br>Wb123: 41% - 35% (-15% change)<br>Bm14: 45% - 34% (-23% change)<br>Bm33: 76% - 55% (-27% change)<br>NIE: 65% - 54% (-17% change)
Poirier 2016	Presumed unexposed (usually mean+/- 3SD)	Cross-sectional cohort:<br>Chikungunya - 75.6%<br>Dengue - 60.6%<br>P. falciparum malaria - 6.3%<br>No evidence of exposure to any pathogen - 13.4%
Priest 2016	Presumed unexposed (usually mean+/- 3SD)	Disease: weighted national estimates (lower confidence - upper confidence)<br>P. falciparum MSP1-19: 4.6% (confidence 3.1 - 6.8)<br>P. vivax MSP1-19: 4.6% (confidence 3.3 - 6.4)<br>Strongyloides NIE: 45.9% (confidence 41.7 - 50.1)<br>Lymphatic filariasis (any two antigens): 2.4% (confidence 1.6 - 3.6)<br>Toxoplasmosis: 5.8% (confidence 4.7 - 7.0)<br>Cysticercosis: 2.6% (confidence 1.8 - 3.7)
Rogier 2017	Presumed unexposed (usually mean+/- 3SD)	PfMSP1-42: 90%<br>PfAMA1: 91%<br>Either or both PfMSP1-42 and PfAMA1: 96%<br>PvMSP1-19: 17.4%
Rogier 2019	Mixture model	PfMSP1: 43.4%<br>PvMSP1: 7.4%

		PmMSP1: 6.7%<br>PfCSP: 16.2%<br>PfAMA1: 25.1%<br>PfLSA1: 7.1%<br>PfGLURP-R0: 11.1%<br>PfHRP2: 11.6%
Tohme 2023	International Standard units	Tetanus long-term protection: 15.1% (95% CI: 14.5 - 15.7)<br>Tetanus full protection: 42.2% (95% CI: 41.2 - 43.3)<br>Tetanus minimal protection: 70.9% (95% CI: 69.9 - 72)<br>Diphtheria long-term protection: 6.0% (95% CI: 5.7 - 6.4)<br>Diphtheria full protection: 41.7% (95% CI: 40.9 - 42.5)<br>Diphtheria minimal protection: 84.3% (95% CI: 83.6 - 85)
vandenHoogen 2021	Mixture model; Other: A two-Gaussian mixture model of log-transformed data, set at the mean +3 SD of the lower distribution.	PmMSP1-19 0-5 years - 7%<br>PmMSP1-19 6-10 years - 9%<br>PmMSP1-19 11-20 years - 18%<br>PmMSP1-19 21-40 years - 28%<br>PmMSP1-19 40+ years - 38%
Won 2018	ROC; Other: ROC curve using sera and presumed unexposed (US citizens)	TAS1 Wb123 - 1.0%<br>TAS1 Bm14 - 6.8%<br>TAS1 Bm33 - 12.0%<br>TAS2 Wb123 - 3.6%<br>TAS2 Bm14 - 3.0%<br>TAS2 Bm33 - 7.8%
Woudenberg 2021	Other: Relative antibody units (RAU) were used in a random forests algorithm to determine seropositivity.	Out of 2415 samples, 174 were classified as positive based on the random forests classification using Luminex measured antibody responses to three SARS-CoV-2 antigens (Spike, RBD, NP). This indicates an overall seroprevalence of 7.2%.
Zambrano 2017	ROC; Presumed unexposed (usually mean+/-3SD); Mixture model	Seroprevalence for intervention group (n=62)<br>VSP3 + VSP5: 41.94%<br>VSP3: 41.94%<br>VSP5: 41.94%<br>Cp17 + Cp23: 37.10%<br>Cp17: 51.61%<br>Cp23: 48.39%<br>p18 + p39: 59.68%<br>p18: 61.29%<br>p39: 72.58%<br>Norovirus (Norwalk) VLP: 42.42%<br>Norovirus (Sydney) VLP: 33.87%<br>Norovirus (St Cloud) VLP: 42.67%<br>LecA: 9.68%<br>ETEC EtxB: 85.48%<br>Vibrio cholerae CtxB: 88.71%<br>Seroprevalence for control group (n=90)<br>VSP3 + VSP5: 31.11%<br>VSP3: 33.33%

		VSP5: 31.11%<br>Cp17 + Cp23: 55.56%<br>Cp17: 71.11%<br>Cp23: 58.89%<br>p18 + p39: 55.56%<br>p18: 57.78%<br>p39: 70.00%<br>Norovirus (Norwalk) VLP: 41.05%<br>Norovirus (Sydney) VLP: 48.89%<br>Norovirus (St Cloud) VLP: 39.47%<br>LecA: 7.78%<br>ETEC EtxB: 80.00%<br>Vibrio cholerae CtxB: 85.56%

S5: Synthesis of data and statistical methods

Author (Year publication)	Synthesis of Data (*Statistical/Model choice*)
**Malaria (two or more species)**	
**Assefa, *et al.* (2019)**	Spatial modelling (*Empirical Bayesian kriging*) for predicted probability; Age-dependent curves (*Reverse Catalytic model*) for seroconversion.
**Byrne, *et al.* (2022)**	Geospatial modelling of predicted seroprevalences to exceedance probabilities (*Matérn covariance function using stochastic partial differential equation approach in INLA*); Seroprevalence by age.
**Byrne, *et al.* (2023)**	Classification of exposure (using *Super Learner algorithm machine learning*); Risk factor assessment (age-adjusted multivariate model); Geostatistical modelling of exposure risks (*Matérn covariance function using stochastic partial differential equation approach in INLA).*
**Feleke, *et al.* (2019)**	Seroprevalence of antigens (*descriptive statistics).*
**Herman, *et al.* (2023)**	Risk factor analysis for seropositivity (*multivariate logistic regression model without random effects for antigen response and Firth's penalized-likelihood logistic regression for PCR-confirmed infections*); Seroprevalence by age; Dynamics in antibodies by age; Correlation of PCR-confirmed infection with seroconversion rates (*serocatalytic conversion model*).
**Jeang, *et al.* (2023)**	Predictors of seropositivity (*Mixed effects logistic regression and multivariate regression*); Seroprevalence by location (*chi-squared and Fisher's exact test* for comparison); Difference in antibody levels (*Wilcoxon rank-sum test*) by location.
**Khaireh, *et al.* (2012)**	Risk factor analysis for seropositivity (*logistic regression model with random effects*); Map of spatial clusters.
**Koffi, *et al.* (2017)**	Seroprevalence by year of sampling and age groups (*Mann-Whitney signed rank test, Spearman rank correction and Fisher's exact test*); Distribution of parasitemia as a function of antibody response (*Generalized linear model*).
**Labadie-Bracho, *et al.* (2020)**	Risk factor analysis (*chi-squared test*); Seropositivity by age and location (*logistic regression*).
**Leonard, *et al.* (2022)**	Geospatial cluster analysis (*Kulldorff spatial scan*) and hot-spot identification (*kernel density estimation*).
**Lu, *et al.* (2020)**	Seroprevalence by age and location; Risk factor analysis (*unweighted odds ratio*).
**Macalinao, *et al.* (2023)**	Seroprevalence by age and location; Difference in antibody levels (*Wilcoxon rank-sum test and Kruskal-Wallis test*) by location; Classification of recent/historical infection (machine learning – "Superlearner"); Correlation of PCR-confirmed infection with antibody responses; Seroconversion and seroreversion rates; Seroconversion curves by antibodies (reverse catalytic model)
**McCaffery, *et al.* (2022)**	Seropositivity by age (*reverse catalytic model*); Seroconversion and seroreversion rates (*likelihood model*).
**Monteiro, *et al.* (2021)**	Reactivity of antibodies by location; Seropositivity by location.
**Oviedo, *et al.* (2022)**	Seropositivity by age and school (weighted K-function); Spatial analysis for autocorrelation (*Moran's I*) and cluster analysis (*Kulldorff's spatial scan*).
**Perraut, *et al.* (2017)**	Seropositivity by age, gender, and location (*Fisher's exact test, Kruskal-Wallis, and Spearman rank correlation test*); Seropositivity by age-groups (*Cochrane-t linear and logistic regression*); Distribution of antibody levels by location; Age-specific seroconversion and seroreversion rates (*reverse catalytic conversion model*).
**Rogier, *et al.* (2017)**	Mapping of seropositivity by school location, and age; Correlation of dynamics between antibodies; Distribution of antibody levels by age.

**Rogier,** ***et al.*** **(2019)**	Comparison of two methods for MBA protocol (k-nearest neighbor-based local regression curves; finite mixture models); Seropositivity by antibody.
**van den Hoogen,** ***et al*** **(2021)**	Dynamics of antibody responses by groups.
**VPDs (two or more pathogens)**	
**Boey,** ***et al.*** **(2021)**	Risk factor analysis for seropositivity (*multiple logistic regression*).
**Breakwell,** ***et al.*** **(2020)**	Seroprevalence by location; Association between seropositivity and vaccine history (*Rao-Scott second order Chi-square test*).
**Cabore,** ***et al.*** **(2016)**	Estimation of population antibody levels (*Geometric mean concentration*) by age and location.
**Feldstein,** ***et al.*** **(2020)**	Seroprevalence by area and age group (compared using *chi-square testing*).
**Khetsuriani,** ***et al.*** **(2022)**	Proportion of population according to antibody levels (*adjusted for sampling response; compared using chi-squared test*); Seropositivity by age and location; Estimation of herd immunity.
**Minta,** ***et al.*** **(2020)**	Seropositivity by age groups and location; National level vaccine coverage estimates; Risk factor analysis for seropositivity (*Rao-Scott chi-square test*).
**Tohme,** ***et al.*** **(2023)**	Seropositivity by location adjusted for sample weights; Risk factor analysis for seropositivity (*Rao-Scott chi-square test*); Mapping of seropositivity estimates.
**Combination of NTDs/VPDs/Other**	
**Aiemjoy,** ***et al.*** **(2020)**	Seroprevalence by age (*classified by stacked ensemble machine learning algorithm "super learner");* Age-dependent antibody curves (*cubic spline for age within a generalized additive model*); Seroprevalence according to distance from nearest water source (*targeted maximum likelihood estimation*).
**Arnold,** ***et al.*** **(2019)**	Serological estimates of force of infection (*S-I-R model, reversible catalytic model and semiparametric spline model*); Age-dependent shifts in population antibody distributions (*semiparametric cubic splines in a generalized additive model*).
**Cadavid-Restrepo,** ***et al.*** **(2022)**	Comparison between antibody and antigen prevalence; analysis by schools/district (*univariate logistic regression analysis*)
**Chan,** ***et al.*** **(2022)**	Risk factor analysis for seropositivity (*logistic regression with random effects*); Geostatistical modelling of exposure risks (*Matérn covariance function using stochastic partial differential equation approach in Integrated Nested Laplace Approximation*).
**Cooley,** ***et al.*** **(2021)**	Estimation of transmission intensity by seroconversion rate (*reversible catalytic model*); Seroprevalence by age.
**Fornace,** ***et al.*** **(2022)**	Geostatistical modelling of exceedance probabilities (10%; 40-60%) for seroprevalence threshold (*Matérn covariance function using Integrated Nested Laplace Approximation);* relationships between estimated seroprevalence and arithmetic mean MFI values per cluster *(B-spline regression).*
**Fujii,** ***et al.*** **(2014)**	Population estimates of seropositivity (*sample weighted probability and finite mixture model*); Distribution of antibody responses (*Kernel density distribution*); Seroprevalence by age and location.
**Kwan,** ***et al.*** **(2018)**	Risk factor analysis for seropositivity (*logistic regression model*).
**Mentzer,** ***et al.*** **(2022)**	Risk factor analysis for seropositivity (*multivariate logistic regression).* Dynamics of antibodies with repeat measurements (*linear mixed model with fixed effects*).
**Miernyk,** ***et al.*** **(2019)**	Risk factor analysis for seropositivity adjusted for age and gender (*multivariate logistic regression*).
**Mosites,** ***et al.*** **(2018)**	Risk factor analysis for seropositivity adjusted for exposure (*multivariate logistic regression*).

**Won, *et al.* (2018)**	Seropositivity by location and time; Difference in seropositivity between repeated measures (*Rao-Scott chi-squared test);* Dynamics of antibodies (*Mood's test*); Mapping of antibody responses.
**Woudenberg, *et al.* (2021)**	Seropositivity by age, location and gender (*chi-squared test*); Seropositivity estimates for population (*Wilson's method*); Dynamics between antibodies and age (*Locally Weighted Scatterplot Smoothing*).
**Zambrano, *et al.* (2017)**	Change in antibodies following intervention (linear regression; t-test with pooled variance estimator); Seroconversion and seropositivity by groups (generalized estimating equation); Age-specific antibody responses.
**Malaria plus combination of NTDs/VPDs/Other**	
**Arzika, *et al.* (2022)**	Change in antibodies following intervention (*mixed-effects binomial model*); age-structured seroprevalence (*semi-parametric proportional hazards model*); Force of infection (*generalized additive mixed model with binomial errors and complementary log–log link*).
**Chan, *et al.* (2022)**	Seroprevalence by age; Dynamics in antibodies by age (*unweighted, two-component finite mixture model*).
**Moss, *et al.* (2011)**	Change in antibodies following intervention (*z-test, Tukey test, Mann-Whitney rank sum, Kruskal-Wallis, and Wilcoxon signed-rank test*).
**Njenga, *et al.* (2020)**	Seroprevalence by location; Age-specific responses (*cross-validated, ensemble machine learning*); Dynamics in antibodies by age (*targeted maximum likelihood estimation with influence curve-based standard errors*).
**Plucinski, *et al.* (2018)**	Seroprevalence by age (*reverse catalytic model*); Estimated of seroconversion and seroreversion; Calculated relative risk outcomes of infection associated with LLIN usage (*Poisson regression*).
**Poirier, *et al.* (2016)**	Spatial hot-spot analysis (*Getis–Ord G statistic*);<br>Seroprevalence by age; Age-group specific antibody responses.
**Priest, *et al.* (2016)**	Weighted national estimates by region and age group (*weighted by non-response adjustment for sampling and regional distribution*); Seroprevalence by age groups and location (*second-order Rao-Scott chi-squared test*).

S6: PRISMA checklist for systematic reviews

PRISMA 2020 Main Checklist

Topic	No.	Item	Location where item is reported
**TITLE**			
**Title**	1	Identify the report as a systematic review.	Title
**ABSTRACT**			
**Abstract**	2	See the PRISMA 2020 for Abstracts checklist	
**INTRODUCTION**			
**Rationale**	3	Describe the rationale for the review in the context of existing knowledge.	Introduction
**Objectives**	4	Provide an explicit statement of the objective(s) or question(s) the review addresses.	Introduction
**METHODS**			
**Eligibility criteria**	5	Specify the inclusion and exclusion criteria for the review and how studies were grouped for the syntheses.	Methods
**Information sources**	6	Specify all databases, registers, websites, organisations, reference lists and other sources searched or consulted to identify studies. Specify the date when each source was last searched or consulted.	Methods and supplementary material 1
**Search strategy**	7	Present the full search strategies for all databases, registers and websites, including any filters and limits used.	Supplementary material 1
**Selection process**	8	Specify the methods used to decide whether a study met the inclusion criteria of the review, including how many reviewers screened each record and each report retrieved,	Methods

		whether they worked independently, and if applicable, details of automation tools used in the process.	
**Data collection process**	9	Specify the methods used to collect data from reports, including how many reviewers collected data from each report, whether they worked independently, any processes for obtaining or confirming data from study investigators, and if applicable, details of automation tools used in the process.	Methods
**Data items**	10a	List and define all outcomes for which data were sought. Specify whether all results that were compatible with each outcome domain in each study were sought (e.g. for all measures, time points, analyses), and if not, the methods used to decide which results to collect.	Methods
	10b	List and define all other variables for which data were sought (e.g. participant and intervention characteristics, funding sources). Describe any assumptions made about any missing or unclear information.	NA
**Study risk of bias assessment**	11	Specify the methods used to assess risk of bias in the included studies, including details of the tool(s) used, how many reviewers assessed each study and whether they worked independently, and if applicable, details of automation tools used in the process.	NA
**Effect measures**	12	Specify for each outcome the effect measure(s) (e.g. risk ratio, mean difference) used in the synthesis or presentation of results.	NA
**Synthesis methods**	13a	Describe the processes used to decide which studies were eligible for each synthesis (e.g. tabulating the study intervention characteristics and comparing against the planned groups for each synthesis (item 5)).	Methods
	13b	Describe any methods required to prepare the data for presentation or synthesis, such as handling of missing summary statistics, or data conversions.	NA
	13c	Describe any methods used to tabulate or visually display results of individual studies and syntheses.	Methods

	13d	Describe any methods used to synthesize results and provide a rationale for the choice(s). If meta-analysis was performed, describe the model(s), method(s) to identify the presence and extent of statistical heterogeneity, and software package(s) used.	NA
	13e	Describe any methods used to explore possible causes of heterogeneity among study results (e.g. subgroup analysis, meta-regression).	NA
	13f	Describe any sensitivity analyses conducted to assess robustness of the synthesized results.	NA
**Reporting bias assessment**	14	Describe any methods used to assess risk of bias due to missing results in a synthesis (arising from reporting biases).	NA
**Certainty assessment**	15	Describe any methods used to assess certainty (or confidence) in the body of evidence for an outcome.	NA
**RESULTS**			
**Study selection**	16a	Describe the results of the search and selection process, from the number of records identified in the search to the number of studies included in the review, ideally using a flow diagram.	Methods and Figure 1
	16b	Cite studies that might appear to meet the inclusion criteria, but which were excluded, and explain why they were excluded.	Figure 1
**Study characteristics**	17	Cite each included study and present its characteristics.	Methods and Table 1-2
**Risk of bias in studies**	18	Present assessments of risk of bias for each included study.	NA
**Results of individual studies**	19	For all outcomes, present, for each study: (a) summary statistics for each group (where appropriate) and (b) an effect estimate and its precision (e.g. confidence/credible interval), ideally using structured tables or plots.	NA
**Results of syntheses**	20a	For each synthesis, briefly summarise the characteristics and risk of bias among contributing studies.	Results

	20b	Present results of all statistical syntheses conducted. If meta-analysis was done, present for each the summary estimate and its precision (e.g. confidence/credible interval) and measures of statistical heterogeneity. If comparing groups, describe the direction of the effect.	NA
	20c	Present results of all investigations of possible causes of heterogeneity among study results.	NA
	20d	Present results of all sensitivity analyses conducted to assess the robustness of the synthesized results.	NA
**Reporting biases**	21	Present assessments of risk of bias due to missing results (arising from reporting biases) for each synthesis assessed.	NA
**Certainty of evidence**	22	Present assessments of certainty (or confidence) in the body of evidence for each outcome assessed.	NA
**DISCUSSION**			
**Discussion**	23a	Provide a general interpretation of the results in the context of other evidence.	Discussion
	23b	Discuss any limitations of the evidence included in the review.	Discussion
	23c	Discuss any limitations of the review processes used.	Discussion
	23d	Discuss implications of the results for practice, policy, and future research.	Discussion
**OTHER INFORMATION**			
**Registration and protocol**	24a	Provide registration information for the review, including register name and registration number, or state that the review was not registered.	Methods
	24b	Indicate where the review protocol can be accessed, or state that a protocol was not prepared.	Methods
	24c	Describe and explain any amendments to information provided at registration or in the protocol.	NA

**Support**	25	Describe sources of financial or non-financial support for the review, and the role of the funders or sponsors in the review.	Conclusion
**Competing interests**	26	Declare any competing interests of review authors.	NA
**Availability of data, code and other materials**	27	Report which of the following are publicly available and where they can be found: template data collection forms; data extracted from included studies; data used for all analyses; analytic code; any other materials used in the review.	Upon request

From: Page MJ, McKenzie JE, Bossuyt PM, Boutron I, Hoffmann TC, Mulrow CD, et al. The PRISMA 2020 statement: an updated guideline for reporting systematic reviews. MetaArXiv. 2020, September 14. DOI: 10.31222/osf.io/v7gm2. For more information, visit: www.prisma-statement.org

S7: Risk Of Bias In Systematic reviews (ROBIS) tool

DOMAIN 1: STUDY ELIGIBILITY CRITERIA
1.1 Did the review adhere to pre-defined objectives and eligibility criteria? Y
1.2 Were the eligibility criteria appropriate for the review question? Y
1.3 Were eligibility criteria unambiguous? Y
1.4 Were any restrictions in eligibility criteria based on study characteristics appropriate (e.g. date, sample size, study quality, outcomes measured)? Y
1.5 Were any restrictions in eligibility criteria based on sources of information appropriate (e.g. publication status or format, language, availability of data)? N
Concerns regarding specification of study eligibility criteria: Low

DOMAIN 2: IDENTIFICATION AND SELECTION OF STUDIES
2.1 Did the search include an appropriate range of databases/electronic sources for published and unpublished reports? Y
2.2 Were methods additional to database searching used to identify relevant reports? N
2.3 Were the terms and structure of the search strategy likely to retrieve as many eligible studies as possible? Y
2.4 Were restrictions based on date, publication format, or language appropriate? Y
2.5 Were efforts made to minimise error in selection of studies? Y
Concerns regarding identification and selection of studies: Low

DOMAIN 3: DATA COLLECTION AND STUDY APPRAISAL
3.1 Were efforts made to minimise error in data collection? Y
3.2 Were sufficient study characteristics available for both review authors and readers to be able to interpret the results? Y
3.3 Were all relevant study results collected for use in the synthesis? Y
3.4 Was risk of bias (or methodological quality) formally assessed using appropriate criteria? Y
3.5 Were efforts made to minimise error in risk of bias assessment? Y
Concerns regarding data collection and study appraisal: Low

DOMAIN 4: SYNTHESIS AND FINDINGS
4.1 Did the synthesis include all studies that it should? Y
4.2 Were all pre-defined analyses reported or departures explained? Y
4.3 Was the synthesis appropriate given the nature and similarity in the research questions, study designs and outcomes across included studies? Y
4.4 Was between-study variation (heterogeneity) minimal or addressed in the synthesis? NA
4.5 Were the findings robust, eg. demonstrated through funnel plot or sensitivity analyses? NA
4.6 Were biases in primary studies minimal or addressed in the synthesis? NA
Concerns regarding synthesis and findings: Low
